# The unravelling of person-centred care: The value and necessity of analysing power relations in contraceptive services

**DOI:** 10.1177/13634593251336175

**Published:** 2025-04-30

**Authors:** Tracy Morison, Catriona Ida Macleod, Yanela Ndabula

**Affiliations:** Massey University, New Zealand; Rhodes University, South Africa; Rhodes University, South Africa; Rhodes University, South Africa

**Keywords:** biopolitics, contraception, contraceptive services, family planning, person-centred, systems

## Abstract

Global research indicates ongoing challenges in delivering person-centred contraceptive care. Much of the contraceptive research investigates this issue using systems-focussed approaches to map institutional constraints (e.g. institutional or health system barriers to accessing contraception). The assumption underlying this research approach is that simply removing structural barriers can address issues and enhance contraceptive autonomy, but this is not the case. Our research shows how discursively constructed power relations undermine bodily integrity and contraceptive agency even as contraceptive providers endorse the principles of patient-centred care. Using a synthetic narrative/discourse approach to analyse provider interviews in South Africa and New Zealand, we draw on Foucauldian analytics of biopower to show how an idealised person-centred care narrative collapses under the weight of discourses of medicalised risk, protectionism, and biomedical expertise, signalling practices of power through confession, responsibilisation and surveillance. Our findings highlight an essential perspective frequently missing in systems-focussed research on contraceptive care: the crucial dimension of power and reproductive politics. Thus, we argue for the necessity of investigating this dimension, in addition to systemic challenges. Our work demonstrates the value of frameworks that illuminate power dynamics, such as the Foucauldian analytics of biopower we undertook. Expanding the range of research perspectives in contraceptive research can deepen understandings of how systems constraints *and* power relations *together* undermine relational person-centred contraceptive care.

The quality of care in contraceptive services became a focal point after the shift to rights-based contraceptive services following the 1994 *International Conference on Population and Development* (Hartmann, 2016 ; Holt et al., 2017). The growing importance of service user- or person-centred care in this sector continued into subsequent decades and remains relevant today (Dehlendorf et al., 2016; Holt et al., 2020). For example, the World Health Organization (WHO) (2022) recently defined one of the competencies of ‘family planning’^1^ healthcare provision as person-centredness, stating, ‘The health worker’s role is to support the individual to make the best decisions possible for themselves. The emphasis is on the individual’s autonomy and right to choose health goals and/or interventions based on identified needs for services.’

Yet, despite three decades of emphasis on quality person-centred care, research still documents problematic healthcare interactions and counselling practices in many settings (Lowe and Rowlands, 2022). Users – especially socially marginalised (cisgender) women – may not be offered the contraception they desire or feel subtly or overtly coerced towards contraceptive uptake or continuance, particularly of long-acting reversible contraception (LARC, Morison and Eagar, 2021). These findings are supported by qualitative research with contraceptive providers, in which participants are found to dismiss service users’ concerns, particularly around side effects (Stevens, 2018); usin persuasive counselling to induce patients to comply with their recommendations, especially in the case of LARC (Berndt and Bell, 2021, 2022; Littlejohn and Kimport, 2017; Mann et al., 2022; Senderowicz, 2019; Senderowicz and Kolenda, 2022; Stevens, 2015, 2018); or feeling that certain people (e.g. young people, people living with HIV) should not be accessing contraception (Hlongwa et al., 2020). These findings collectively highlight the limitations on service users’ contraceptive agency and person-centred care more broadly, suggesting that it remains an unrealised ideal in many contexts. At the same time, however, participants readily acknowledge systematic constraints, while emphasising their commitment to person centredness (Berndt & Bell, 2021; Morison, 2023).

This finding aligns with broader qualitative research on service provision in which clinicians readily identify ‘the system’ as an obstacle to service delivery (Waring, 2007). Likewise, contraceptive providers point to institutional or health systems challenges as the barriers to quality person-centred contraceptive care, as noted in a recent systematic review of research on providers’ perspectives of contraceptive care. These barriers include insufficient time, inability to offer same-day method insertion, and a lack of training (Manzer et al., 2024). Accordingly, researchers recommend systemic changes to ensure better provision of contraceptive care. For example, Lince-Deroche et al. (2020: 1) propose ‘addressing staff workloads and providing refresher training on contraception’ based on their study of contraceptive provider perspectives on service delivery in South Africa – one of our study sites. Similarly, in our other study site, Aotearoa (New Zealand), McGinn et al. (2019: 63) reported that ‘robust primary care training and funding for contraception will be required’ to overcome barriers to LARC provision.

Although addressing such institutional or health systems constraints is indeed essential for strengthening person-centred care, this alone is insufficient. Much of the existing contraceptive research assumes that contraceptive autonomy can be improved solely by removing structural barriers. While such systems-focussed approaches effectively map institutional constraints, they are limited in their capacity to address normative questions and illuminate the discursively constructed power relations shaping care practices (Stojanovic et al., 2016). As a consequence, researchers often fail to attend to power relations, including how even person-centred practices may have – as Gerrits (2014: 125) points out – ‘unintended disciplining and normalising effects’.

In light of this oversight, our aim in this article is to explore an essential perspective often overlooked in systems-focussed research: the biopolitics of contraceptive care. Using data from a transnational study of LARC provision and use in South Africa and Aotearoa, we seek to illuminate the complex processes through which power relations that impede person-centred care operate and are reproduced. We analyse contraceptive providers’ accounts of their experiences of delivering care, specifically, how providers describe interactions that fail to promote contraceptive agency, which we conceptualise as ‘the socio-culturally mediated ability to act and decide on contraception’ (Morison et al., 2022: 229). Our findings shed light on the power relations that undermine person-centred practice and thus underscore the importance and value of attending to power relations in thinking through person-centred care. We argue that, in addition to identifying systemic challenges, understanding how power relations are created and maintained in diverse contexts is crucial for expanding conceptualisations of person-centred care – and ultimately enhancing care. Our findings offer critical focal points for further inquiry and transformation of these dynamics.

We follow Gerrits (2014) and Cook and Brunton (2015) in drawing on Foucauldian analytics of power to highlight the matrix of interactive, discursive, and social power relations embedded in reproductive healthcare interactions. We focus on what Foucault (1963/2003) termed ‘bio-power’: the intertwined regulation of the individual body and the social body politic. Through bio-power, ‘life and its mechanisms [are brought] into the realm of explicit calculations . . .[making] knowledge-power an agent of the transformation of human life’ (Rabinow, 1984: 266). Reproductive health – an essential aspect of ‘life and its mechanisms’ – intertwines (i) population-level knowledge (e.g. epidemiologies of unintended pregnancies, ‘unmet need’ for contraception, etc.); (ii) rationalities regarding the regulation of health, mortality, morbidity, and families (e.g. the Sustainable Development Goal of reducing maternal mortality); (iii) the clinical gaze (Foucault, 1963/2003) that sees and knows the reproductive body (and thus the best clinical remedy); and (iv) disciplinary technologies that incite healthcare providers and users to produce efficient, self-disciplined reproductive bodies. Disciplinary technologies refer to the subtle, systematic, and institutionalised mechanisms through which power relations are enacted. Disciplinary practices, such as surveillance, normalisation (defining and contrasting the normal and abnormal), and the confession shape interactions and behaviour without the need for direct oppression.

Before we go on to describe our study, a caveat is needed. Although person-centred care is widely promoted as a means to empower patients and reduce hierarchical dynamics, it remains a contested concept. Recognising critiques that the focus on individual autonomy can inadvertently reinforce neoliberal and individualistic paradigms and reproduce power imbalances, we draw on Buetow’s (2016) relational perspective of person-centred care. Rather than a fixed, normatively ‘true’ approach we conceptualise person-centred care as a dynamic process that negotiates the inherent power relations between providers (as ‘givers’) and service users (as ‘recipients’). From this perspective. Person-centred contraceptive care involves relational and inter-subjective healthcare relations –acknowledging the personhood of *both* service users *and* providers (Buetow, 2016) and that these relations are shot through with socially embedded discursive practices and power relations. This relational understanding also acknowledges both the emancipatory potential and the limitations of person-centred care in practice.

In the context of contraceptive services, we understand person-centred care as a reflexive and interactional process in which the provider-as-person recognises and fosters the user-as-person’s bodily integrity and decision-making agency in using/not using (particular types of) contraception. Reflexivity ensures that both user agency and person-centred care are understood as simultaneously enabled and constrained by both institutional or health systems issues and discursively constructed power relations. With this in mind, we investigate contraceptive providers’ accounts of interactions that fail to uphold a person-centred discourse and, ultimately, to promote contraceptive agency, homing in on power relations.

## Our study

Our data are drawn from a transnational study on LARC provision and use in South Africa and Aotearoa. In this article we focus on contraceptive providers’ accounts of their experiences of delivering care in these two contexts. Both countries share a history of racially motivated contraceptive provision. Class-based and racialised patterns in service delivery are rooted in colonialism, White nationalism, and, from the turn of the 20th century, eugenics (Hartmann, 2016). The broader backdrop is, of course, as Ratangee (2023: 23) argues, that ‘Medicine allowed colonists to set foot in developing regions and then assert their power and authority, fuelling the institution of imperialism itself.’ The colonial introduction of Western biomedicine, with its understandings of clinical care and health that continue to shape power dynamics in clinical care (Graham & Masters-Awatere 2020, Pelzer 2009).

Concentrating on these two contexts aligns with the transnational feminist notion of ‘chains of equivalence’ (Mohanty, 1999): the contextual commonalities across different places (and by implication the specificities of each). Racialised and class-based inequities persist in contraceptive care in both countries, despite different economic trajectories.

South Africa is a middle-income country with stark inequality, epitomised by its bifurcated public-private health system (Lince-Deroche et al., 2016, 2020). The country has rights-based, enabling health policies and high contraceptive knowledge, but health service access and use are shaped by wealth disparities. A severely under-resourced public sector is used by the poor (largely Black) majority, while the middle-class minority pays for private services. Private services often provide more method options than public-sector clinics, which typically only stock pills,external (‘male’) condoms, and injectable contraception; intra-uterine devices are frequently only available in urban areas and at referral facilities (Holt et al., 2012; Lince-Deroche et al., 2016, 2020). Commodity stock-outs or limited access are common complaints among poor and rural people (Hlongwa et al., 2020). In the public sector, injectables are the most used and readily accessible form of contraception, other than external condoms. In comparison, subdermal implants and modern intrauterine devices (IUDs) were introduced relatively late in South Africa and are thus less widely used than more familiar methods (Pleaner et al., 2017).

Aotearoa is a high-income nation, where reproductive politics are still shadowed by racist legacies (Cram et al., 2024; Beddoe, 2014; Huria et al., 2023). Overall, there is better access to most contraceptive methods than in South Africa, including LARCs. Long-acting methods are available for free or at low cost through primary public healthcare and some specialist non-governmental agencies. There are associated costs, like surgical and GP appointment fees, which vary across providers. Costs and service availability are variable due to system challenges, including resource allocation and skilled worker shortages. These factors, alongside entrenched racialised health inequities, disadvantage marginalised women: those on low incomes, in rural or remote areas, and from marginalised ethnic groups (McGinn et al., 2019, 2021). The national sexual and reproductive health policy is largely neoliberal and risk-focussed, classifying Maori, Pasifika, and youth as ’at-risk’ groups (Morison and Herbert, 2019). Against this backdrop, LARC has gained popularity, sometimes presented as a solution to social issues like welfare ‘dependency’, teenage pregnancy, and poverty and historically targeted ‘at risk’ women – primarily low-income, young and/or ethnic minority women (McGinn et al., 2021). The latest Ministry of Health (2020: 6) guidance takes a LARC-first approach, stipulating that ‘LARC should be considered a first-choice contraceptive.’

## Methods

We used a site-based approach to recruit contraceptive providers in each country, following ethics clearance from our respective universities (MasseyUniversity; Rhodes University). Tracy and Yanela shared calls for volunteers via email invitations and electronic or physical advertisements distributed by relevant healthcare organisations in Aotearoa and South Africa, respectively. Yanela also gave presentations about the research to providers at healthcare organisations. Referrals were used in both settings, as participants referred interested colleagues to us. A total of 43 participants were recruited: 21 providers from 14 sites in South Africa and 22 providers from 16 sites in Aotearoa, as summarised in Tables 1 and 2.

**Table 1.** Participant characteristics.

Characteristics	South Africa (21)		Aotearoa New Zealand (22)	
Gender	Female	19	Female	19
	Male	2	Male	3
Race/ethnicity	Black (Xhosa)	16	Indigenous (Maori)	4
	White (Settler)	3	White (Immigrant & Pakeha^a^/Settler)	18
	Coloured^b^	2		
Role	Nurse	17	Nurse	7
	General practitioner	2	General practitioner	1
	Specialist	1	Specialist	5
	Intern	1	Nurse practitioner	3
			Community health/social worker	2

a Pakeha is a Maori term for non-Indigenous New Zealanders of settler-European descent.

b This racial category describes people of mixed race, descended from the indigenous Khoisan and other African or Asian peoples, many of whom were enslaved at the Cape colony.

**Table 2.** Overview of healthcare settings (30 sites).

Setting		South Africa (14 sites)	Aotearoa New Zealand (16 sites)
Urban	City	4 Public clinics and 1 hospital	2 Specialist sexual & reproductive health providers (NGO)
		1 Private GP practice	
	Regional city	5 Public clinics	3 Public hospitals
		1 Private GP practice	8 Public clinics
	Town	–	2 Public hospitals
			1 Public clinic (school)
Rural	Village	2 Public clinics	–

The participants took part in an in-person (*n* = 40) or telephonic (*n* = 3) narrative interview about general contraceptive care practices and experiences (counselling; methods most/least recommended, to whom, and why) and long-acting reversible contraceptive (LARC) provision (type most/least recommended, to whom, and why; eligibility criteria; memorable cases). All the interviews in Aotearoa were conducted in English by the first author, Tracy, a White woman of European descent. In South Africa, the interviews were conducted in a combination of English or Xhosa (an Indigenous language) by the third author, Yanela, a young Black Xhosa woman. She and two bilingual transcribers/ translators translated Xhosa into English for analysis.

The data were analysed using the Foucauldian methodologies proposed by Barker (2017) and Parker (1992). Drawing on Foucault and Ricoeur, Barker (2017) employs a synthetic methodology for analysing talk. As such, the analysis combines the post-structuralist interest in the macro elements of discourse (e.g. socio-cultural context) with attention to narratives as a form of social action. In this approach, discourse refers to a coherent way of speaking about and representing the world that constructs it in particular ways. It comprises a unified system of meanings that pre-exist talk about a topic or issue (e.g. a discourse of personal responsibility frames contraception as an individual obligation) and delimits what is sayable and doable in relation to it (e.g. non-use of contraception denounced as irresponsible or deviant) (Burr, 2024).

Barker (2017: 399) refers to the representation and constitution of different ways of being and doing through the process of ‘emplotment’ in narration. Speakers emplot culturally available discourses into contextually relevant narratives as ‘sites where social structures (discourses, . . .power relations), material conditions, and individuals meet and interact’ (Mavuso et al., 2019: 18). Thus, for Barker (2017: 409), the subject ‘is both highly constrained by discourse . . . but not fully determined by it’. A narrator actively and reflexively uses discourses in the ‘active, interpretative process of emplotment’ (399) to achieve particular rhetorical purposes. For example, a biomedical expertise discourse may allow healthcare providers to justify curtailing patient involvement in decision-making by positioning themselves as experts advising uninformed patients.

Analytically, Barker’s (2017) approach comprises three iterative tasks: (1) identification of narratives, (2) exploring the emplotment of discourses within these, and (3) attending to the rhetorical purposes of emplotment. We began by identifying narratives related to providing contraceptive care, which are signalled by talk containing a sequence (temporal ordering) and/or sense-making about events or people (explanations, ascriptions of causality). For each, we looked for discourses emplotted within, that grant narrative intelligibility. For this we used Parker’s (1992) seven basic criteria for identifying discourses, namely, (i) discourses are realised in text, (ii) are about objects, (iii) contain subjects, (iv) are a coherent system of meanings, (v) refer to other discourses, (vi) reflect on their way of speaking and (vii) are historically located. We coded patterned language use (e.g. pattern of familiar themes, motifs, or truisms; recurring words/phrases, imagery, metaphors etc.) that point to a coherent system of meanings that pre-exist talk about a topic or issue and delimits what is sayable and doable in relation to it (Burr, 2024). We then considered each discourse contextually to establish how it is ‘put to work’ (though emplotment) to particular ends, enabling specific subject positions and social actions.

## Analysis and discussion

Below, we show how a person-centred discourse was emplotted (or drawn on) by providers in the narrative ‘They decide for themselves’. We then discuss the three narratives in which person-centredness unravelled, revealing particular power relations that render user agency ‘unrealistic’ or ‘foolish’. These are: (i) ‘It depends on what is available’, which emplots a discourse of institutional/structural constraints; (ii) ‘To reduce your risk’, which emplots discourses of bio-medicalised risk and protectionism, and (iii) ‘There is often misunderstanding’, which emplots the discourse of bio-medicalised expertise.

### ‘They decide for themselves’: Person-centred discourse

A person-centred discourse *was* drawn on by providers in some of their talk. They emphasised users’ deciding ‘for themselves’ what contraception they would use – after being provided with information. For example:

**EXTRACT 1**

**Thami (public health nurse, SA):** We inform them of all their options, and they decide for themselves. Then, we inform them thoroughly how each and every one of them works. If they find that the one they chose is giving them issues, they have the right to change it and start with a new one and then see how that one treats them. They don’t have to stick to one method when it doesn’t work for them.

**EXTRACT 2**

**Claire (nurse, public youth-friendly clinic, NZ):** Well, all of our contraception is offered with informed . . . consent. [. . .] We spend time teaching people about it and showing them the options and talking about the pros and cons realistically [. . .] And so, if you’re not happy with your implant, you must come back and talk about it, and you don’t have to keep it. We can take it out.

A person-centred discourse is evident in the construction of the provider role as providing ‘thorough’ information to enable a service user to make an informed, autonomous decision. In this narrative, ‘They decide for themselves’, the user has the right to decide on the method or change it. The provider’s role is simply to facilitate the decision (‘inform them’; ‘spend time teaching’). Such talk was not uncommon across the datasets. It was clear that a person-centred discourse has filtered into the narratives of service providers in these two countries. The assumptions of a person-centred approach – as detailed by the World Health Organization and other policy frameworks – are known and reiterated by providers.

However, while present, this discourse started to unravel as providers narrated their experiences, as shown in the following extracts.

**EXTRACT 3**

**Nozi (nurse, public clinic, SA):** We recommend all of them [all contraceptive methods], but the ones that are mostly used are the injectables and your oral and your *Implanon*. . . . The least would be the loop [IUD]. /Yanela: People don’t usually ask for it?/ Not necessarily that they don’t want it, it’s just that it is new to us, because (.) there was one [nursing] Sister who went to training on how to insert it, because it was usually doctors that inserted it.

**EXTRACT 4**

**Marama (nurse – non-profit youth-friendly health centre, NZ):** We wanted our GPs to kind of help out and get us trained, but the contract doesn’t allow. We didn’t have the funding here to release some doctors over [to the healthcare centre]. [. . .] Lots of people want to get something long term and [. . .] *Mirena* [intrauterine system] is quite a preferred option, but these kids can’t afford it. So we go, ‘Oh, you can have the copper one, because that’s funded’. But hold on! We’ve got no one to put it in. ((Laughter))

Here, Nozi and Marama discuss the skills and funding shortages that deter them from practicing person-centredness. Users may ‘prefer’ or ‘want’ a particular method, but since no service provider is trained to administer it or there is no money to pay for it, it becomes a non-option. User agency is, thus, severely curtailed. These extracts highlight a common issue across data from both countries: regulations governing who may insert implants and intrauterine methods. These minor procedures are often limited to doctors to perform, with disputes and complications around allowing midwives or nurses to administer them (McGinn et al., 2019, 2021; Pleaner et al., 2017).

As explained below, three narratives highlight how a discourse of person-centredness unravels in provider-user interactions. The first narrative draws from a discourse of institutional/structural constraints (referred to in the above extracts), while the discourses of medicalised risk, protectionism, and biomedical expertise underpin the second two.

### ‘It depends on what is available’: Institutional/structural constraints discourse

Participants frequently emplotted a discourse of institutional/structural constraints when discussing their work settings, including funding and resource shortages, unsupportive or restrictive institutional arrangements, structures, or policies. These were depicted in the narrative, ‘It depends on what is available’, as limiting what was possible in terms of providing contraceptive services to patients, sometimes even as preventing them from providing the sort of care they believed was best quality (e.g. longer consultations, follow-up care). For instance:

**EXTRACT 5**

**Jason (general practitioner, SA):** Um, [in terms of] the long-acting ones most women go for the injectables. ((Explains different types, duration of efficacy.)) It also depends on what is available. We have had times where we sort of had stock issues and that sort of thing and you have had to order [. . .] I haven’t had too many *Implanon* [implants] since when I was in [the] government [sector] [. . .] Very few [implants] in government. [For] *Mirenas* [intrauterine system, IUS],^2^ we always had to have [a] motivation for the government to supply [them], because they are costly. So, we offered quite a lot of *Implanon* and injectables mostly.

**EXTRACT 6**

**Tina (nurse practitioner – women’s health clinic, NZ):** If they had a community services card and they were low-income, on the DPB [Domestic Purposes Benefit], we would be able to get some funding through *WINZ [Work and Income New Zealand]* and that’s all stopped. That’s only recently, though. [The] people that we were offering it to, we can’t anymore. So, we don’t. We don’t have that option, because if I can’t afford it, [and] if they can’t pay, then we can’t do it.

**EXTRACT 7**

**Janet (Gynaecologist, public hospital, NZ):** We’ve got one particular practice where they have actually minimised the cost [of providing LARC], but I think they’ve also minimised the time for the consultation. So, we did get feedback from one woman that she felt a bit shell-shocked during the procedure. I think it’s just she wasn’t well prepared for it because they’ve cut it to the bare bones [so,] they can actually offer it a lower cost. [. . .] There is some funding now. [We’re] trying to streamline it now and get it to the people who need it most. The *Mirena* [intra-uterine system], of course, has been very middle-class, because of the costs involved.

These extracts highlight how access to contraceptive care is bifurcated along class-based lines, which are racialised in both countries: ‘middle-class’ (extract 7) versus ‘low-income’ (extract 6) patients using ‘government’ (extract 5) services. Those reliant on government-subsidised contraceptive services are positioned as most affected by funding constraints, with fewer choices available to them than those able to afford private care. While these material constraints shape the clinical environment, our analysis primarily examines how providers discursively construct these conditions to justify particular care practices. For example, a narrative of choice (‘women go for the injectables’) devolves into one of constraint (‘we always had to have a motivation’; ‘people we were offering it to, we can’t anymore’; ‘cut to the bare bones’). An institutional/structural constraints discourse, thus, foregrounds structural restrictions on the autonomy of contraceptive users. A person-centred discourse is not abandoned, however. For example, in extract 7, Janet emphasises attempts to increase access to a broader range of contraceptive methods, especially for those ‘who need it most’ (i.e. have been previously disadvantaged).

### ‘To reduce your risk of unintended pregnancy’: Medicalised risk and protectionism discourses

Throughout the data, participants assumed unintended pregnancy to be a highly undesirable, adverse outcome. As such, a medicalised risk discourse was emplotted to construct a narrative in which unintended pregnancy should be avoided at all costs through reliable, responsible contraceptive use – use that should be facilitated by healthcare providers whose expertise puts them in a position to understand and protect users from irresponsible use and risk. The following extracts offer illustration.

**EXTRACT 8**

**Carol (public clinic nurse, SA):** /Yanela: . . .which ones do you recommend the least?/ The least? We do look at your reliability in all the type of things, and what type of person you are. Like, are you a very active, like a busy person? Like [. . .] for the postnatal moms that have just delivered, what happens from past experiences is that they usually forget, because the babies are so small, the babies are, it’s hectic in the beginning getting a [. . .] a routine in place. So, they tend to forget the pill, and then they become pregnant soon after. So mainly we’ll promote, not promote, but we’ll tell them that the injectable is a bit better than the tablets.

**EXTRACT 9**

**Gavin (public sector Gynaecologist, NZ):** Ideally you want to wait three to four weeks [after a woman has given birth] before giving the *Depo [Provera* injection] to reduce your risk of irregular bleeding afterwards, but there are always going to be women who (.) get nervous because of their social background, education level. They’re not going to rock up and have their *depo* given three weeks later. So, we would give it, you know, while they’re on the ward. So, we know that at least for the next three months they are protected, and hopefully the midwife can sell the message that she [the patient] needs to continue being seen by GP to have the injections. So, you can see immediately the advantage of the *Jadelle* [implant].

In these extracts, constraints talk shifts from the institutional and structural context to the individual. In both Carol’s and Gavin’s narratives, post-partum women themselves are the problem: they are at risk of forgetting or failing to return for contraception. Characterising service users as both at-risk *and a risk* constructs some as ideal candidates for LARC (Bertotti et al., 2021), as evident when the speakers refer to injectable contraception as best for postpartum women. Indeed, providers generally constructed high-efficacy contraception (LARC) as the best option for keeping women safe from unintended pregnancies but, more importantly, from their own irresponsibility. Thus, an injunction to avoid unplanned pregnancies interweaves with a medicalised risk discourse, focussing on potential ‘exposure to the risk of conception’ (Higgins, 2010: 153).

In this scenario, the provider’s primary task is to ‘protect’ women from themselves and their unintended pregnancy risk by *ensuring* contraceptive use. The participants depict their actions – promoting or imposing injectables – as pre-empting women’s unreliability. The service users’ implicit failure to ensure ongoing contraceptive use creates a sense of urgency and warrants actions curtailing agency. For instance, in extract 9, pre-emptively administering LARC to certain, potentially problematic women ‘while they’re on the ward’ is deemed opportune, with potential self-imposed risk warranting urgent action. While framed as protective measures, such strategies reflect a paternalistic stance wherein providers assert their authority over patients’ reproductive decisions, ultimately reinforcing the hierarchical dynamics embedded in biomedical practices (Morison, 2022; Stevens, 2015). Such talk echoes the sentiment that to ensure contraceptive use, especially of LARCs, providers should ‘strike while the iron’s hot’ (Lou, midwife), even if it means curtailing service users’ agency. In this vein, Gavin (extract 9) also construes implants as having an ‘advantage’ over other methods due to its long-acting and, implicitly, imposable nature.

Two additional points are noteworthy in the extracts above. First, the explanations for postpartum women’s lack of reliability are different. Carol’s narrative locates new mothers’ forgetfulness around contraception within the stresses of caring for a newborn baby, rendering it understandable, though the women are nevertheless still deemed unreliable. Gavin, however, indicates that the problem lies with the women’s personal characteristics (‘social background, education level’), which may make them ‘nervous’ to follow up. While both explanations justify providers initiating protective interventions (in particular, prescribing LARC), Carol’s explanation can apply to all postpartum women, whereas Gavin’s focuses on particular ones. Second, both Carol and Gavin recognise that LARCs may not be a users’ first choice. Carol speaks of ‘promoting’ the injectable, catching herself (‘not promoting’) because of the possibility of being positioned as coercive. Gavin’s wish for midwives to ‘sell the message’ suggests that although evident in their narratives, provider protectionism must be negotiated in situ, with providers having to deploy various persuasive tactics (Morison, 2022).

The singling out of at-risk groups of women is enabled through the disciplinary practice of surveillance (Clarke et al., 2003). Carol’s surveillance is personal: she ‘look[s] at your reliability in all the type of things, and what type of person you are’. Gavin, on the other hand, relies on broad-brush strokes: people ‘get nervous because of their social background, education level’. This surveillance feeds into dividing practices in which certain women are seen as requiring special attention/protection and ‘in need of more disciplinary and invasive technologies of biomedicalisation’ (Clarke et al., 2003: 184).

This surveillance and the accompanying dividing practices, whereby particular ‘vulnerable’ groups are singled out, were often class-based, and intersected with racialised motifs as shown in the following examples.

**EXTRACT 10, (Mesani –public clinic nurse, SA):** Why [they won’t use family planning]? I ask them why, and they don’t know. It’s because some other men have the myth that when you’re using family planning, especially those who have already have children, they are going to [experience a] loss of libido. Like, in Xhosa, they say, They are ‘cold.’ They are not as ‘hot’ as they used to be when they’re using family planning. They’ve got that myth. With the Coloureds,^3^ they always say, ‘No, we want more kids, so don’t use family planning, so don’t inject’.

**EXTRACT 11, Gavin (Public sector gynaecologist, NZ):** So, I think the *Mirena* is fantastic for people (.) the *Mirena* or the *Jadelle*, for people from lower socio-economic backgrounds. [. . .] It’s not so common for European women to go away with no contraception. Pacific Island woman, Maori woman yeah, yeah, they might. They, they may, and they’re kind of highest risk group, really.

In Extract 12, Mesani dismisses embodied concerns (like loss of sexual desire) as culturally located myths associated with specific racialised groups. This implies that addressing people’s resistance to contraception (‘I ask them why’) requires providers to orient to these groups’ cultural beliefs, such as libido myths amongst Xhosa people and proper family planning amongst ‘Coloured’ people. In Extract 11, ascribing contraceptive irresponsibility to ‘lower socio-economic backgrounds’ shifts seamlessly to implicating race, with Maori and Pacific Island women singled out as the ‘highest risk group’. Such talk was, however, most often subtle. Several participants used ‘coded language’ (Manzer and Bell, 2021: 127) concerning unreliable service users to invoke implicit shared understandings of race . For example, in South Africa, Lynn (gynaecologist) referred to ‘culture’ or people from ‘the township’ (urban areas reserved for Black people during apartheid); in Aotearoa, Pippa (nurse), Gavin (Gynaecologist), and Renee (nurse) spoke about ‘culture’.

In terms of those at risk, teenagers and young women were frequently positioned as unreliable across both settings, as noted by Mann et al. (2022) in their recent US study. For instance:

**EXTRACT 12**

**Mesani (public clinic nurse, SA:** You must meet them halfway because they don’t know, and they are children. [. . .] And I say, ‘What do you say?’ [. . .] ‘No, I want [the] injection’. ‘Why?’ ‘My mother said I always go out with friends, and I come home late’. And then I say, ‘Do you have a boyfriend?’ [. . .] ‘No, sister, I don’t have a boyfriend’ And then I say, .‘Your mother, why, when you’re coming home late? Tell me the truth.’ And then you must try and be fair here. ‘Tell me the truth so that I can help and explain to you’. ‘Yes, Sister, I have got a boyfriend’. ‘Did you sleep with your boyfriend?’. And then there are those who are bluffing, ‘Once, sister’. ‘No, Sister; once, Sister’. And then you must start educating and explaining.

**EXTRACT 13, (Michelle – nurse practitioner, NZ):** Maybe a young person that comes in—possibly with an adult—and the young person might be really keen to have the pill and so we talk about, you know, you need to be able to remember to take it on time, and the adult will say ‘They’re not gonna. . . I don’t think they’re going to remember’. So, it’s kind of talking about that.

In these extracts, the participants position young people as unreliable: misrepresenting their sexual history (Extract 12) and failing to comply with the daily regimen required for the contraceptive pill (Extract 13). The disciplinary practices of confession (‘tell me the truth’) and responsibilisation (‘remember to take it on time’) are deployed to underline the need for additional ‘educating and explaining’ by the provider. Medicalised risk and responsibilisation work together here to implicitly render young women as ‘at risk’ for unplanned pregnancy based on the Western, class-based ideal of delayed motherhood (Stevens, 2015). This risk-saturated, irresponsible subject position is reinforced by the prevalent view of ‘teenagers’, with its dominant ‘understanding of young people as risk takers, as well as young people at risk due to inherent characteristics—their inexperience, [and] biological factors’ (Montero and Kelly, 2016: 53) derived from developmental psychology. This view is exemplified by Erika’s? (midwife, Aotearoa) explanation that some young women fail to contracept because they ‘just can’t actually be bothered’.

In addition, young women judged as lacking in responsibility or capacity are not only positioned as *at risk* of unintended pregnancy, and in some cases *as a risk* themselves, jeopardising not only their wellbeing but that of their potential offspring and broader society (Lowe, 2005). Allusions were made to both these risks, but particularly to wider society, reflecting a public health framing in which LARC features as the ideal solution. For instance, there was talk of ‘huge numbers of unplanned and unwanted pregnancies’ (Messani, public health nurse, SA), teenage pregnancy as an ‘ongoing cost to society’ (Gavin, Public health gynaecologist, NZ), and emphasis on the cost-effectiveness of LARC in public health (Jason, general practitioner, SA). These risk-based understandings warrant providers’ inability to trust young women, as they are likely to ‘default’ (Thandisiwa, nurse, SA) on appointments or lack the motivation to use contraception reliably.

### ‘There is often a misunderstanding’: Biomedical expertise discourse

The participants commonly emphasised the authority of expert knowledge in defining concerns and priorities in contraceptive decision-making, simultaneously diminishing, and even disparaging, alternative or competing issues that service users bring to consultations.

**EXTRACT 14**

**Lynn (public sector gynaecologist, SA):** Usually, the sorts of things that people are asking is, ‘My friend fell pregnant on the loop [IUD]. I don’t want the loop.’ Um. ‘Somebody lost the loop.’ There is often a misunderstanding of how things work. They don’t want the Implanon [implant]. I have heard very interesting things about why people want the Implanon removed. ‘My arm is weak since I have had the *Implanon* inserted’, and, yes, fair enough, if it’s inserted deep into the muscle or something, perhaps. But this [implant] was very much in the correct position. So, it’s all those things. [. . .] With the IUCD [Intrauterine contraeptive Device]?, it’s: ‘The baby came out holding it. I fell pregnant.’ ((Laughter)) I’m like, ‘No, the baby wasn’t holding it! Someone made that picture up and posted it [online]. It’s not true.’

**EXTRACT 15**

**Marama**^4^ **(Youth-friendly, non-profit health centre, NZ)**: It’s nutting out all those myths and once you actually do that, then they’re more inclined to wanna [Melanie: Be open] be open, to give it a shot. Especially with the *Jadelles* [implants]. They hear these [stories], ‘Oh,’ you know, ‘the bleeding and the surgical procedure is. . .’ To them, that sounded very horrible, but once you really nut it out and sit down, they’re like, ‘Oh, actually, I will give it a go.’ You know? So, selling. . . trying to sell more of the pros about it, than the cons. You know? I mean, obviously, as nurses, we have to discuss the side effects and all that kind of stuff, but not making it sound like it’s a real horrible thing for any of them. [. . .] So, I’m always trying to be real open and honest but, you know, I try and push to more of a long-term contraception.

Here, service users’ reasons for declining specific methods, requesting reversal, or reporting side effects are derisively attributed to ‘very interesting’ reasons and unfounded ‘myths’and delegitimised. To make her case, in Extract 14, Lynn uses an outlandish example: a sensationalist Internet meme of an infant born holding an intrauterine device, thereby effectively dismissing patient concerns. Service users are positioned as more than simply as lacking the knowledge and expertise to understand medical information but as gullible and foolish. This positioning is reinforced by the obviously disparaging tone and the collective laughter.Likewise, other participants used hyperbolic language and extreme case formulations—such as reference to ‘really bizarre,’ ‘horror stories’ (Logan, midwife, NZ)—that render service users’ concerns unworthy of being taken seriously. The tendency to delegitimise service users’ experiential knowledge not only underscores the reliance on a biomedical expertise discourse but also reveals the clinical paternalism inherent in this discourse, which positions providers as the ultimate arbiters of what constitutes valid health information (Stevens, 2015).

The extracts above illustrate how service providers disregarded (certain) service users’ experiential, embodied, or cultural knowledge in contraceptive decision-making frequently portraying their concerns about contraception as misguided or based on ‘misunderstanding’ (extract 14), and so often leading women to make poor choices about contraception. Such talk sometimes took on a disparaging tone, positioning service users as irrational and unreasonable patients and, hence, in need of responsibilisation.

Ultimately, drawing on a biomedical expertise discourse positions the provider as uniquely able to judge valid concerns and works to dismiss service users’ reports as medically implausible. Accordingly, service users’ reports could be discredited and dismissed, firstly, as unreasonable ‘complaints’—which were sometimes implictly linked to unreasonable expectations and/or inadequate ‘education’—or, secondly, as overreactions to side effects that were described as rare, temporary, or mild (‘just a little bit irregular’). Such minimisation is also reported in several international studies (Amico et al., 2017; Littlejohn and Kimport, 2017; Mann, 2022; Manzer and Bell, 2021, 2022; Stevens, 2015, 2018). The biomedical expertise discourse allowed issues that service users bring to consultations to be judged as discreditable and dismissible and for providers to prioritise the public health goal of pregnancy prevention—and hence method efficacy– over and above other concerns or issues raised by service users. As Berndt and Bell (2021: 621) conclude, ‘for providers, then, side effects are often ephemeral and tangential to the essential components of contraception – efficacy and ease of use. Providers prioritise these components, with this end justifying most means.’ Thus, contraceptive decision-making is rendered a factual endeavour involving the rational calculation of costs versus benefits, with side effects simply a cost of pregnancy prevention (Stevens, 2018). This sentiment was common among our participants; one even suggested that side effects associated with LARCs be renamed as ‘expected effects’ (Erika, midwife, NZ) to temper unrealistic expectations and increase adherence, rendering providers’ focus on pregnancy prevention entirely reasonable.

## Concluding discussion

Our participants endorsed the ideal of person-centred care in policies and training, stipulating that users ‘must decide for themselves’. Yet, despite their explicit endorsement, their talk also demonstrates how person-centred contraceptive care unravels in the context of *both* system constraints *and* discourses of medicalised risk/protectionism and biomedical expertise. Thus, person-centred care and, ultimately, contraceptive agency are undermined by insufficient resources (commodities, time, human resources, training) alongside the instantiation of power imbalances through constructions of ‘vulnerability’ and risk and the simultaneous paternalistic privileging of providers’ biomedical knowledge and denigrating of users’ embodied knowledge.

The first set of issues, systems constraints, are typically the focus of research in this area. This work concentrates on institutional and health systems ‘barriers’ and making recommendations focussed on removing these through, inter alia, better supply chain management, ensuring stocking of the full suite of contraceptive methods, training in person-centred care, and providing sufficient personnel and resources in healthcare facilities. This ‘barrier-type’ research may also focus on interpersonal or organisational dynamics, such as discrimination. For example, Holt et al. (2020: s880) indicate that quality services should include attention to ‘equity and racism’ and ‘equitably respect and meet individuals’ needs,’ recommendations reflected in the WHO’s (2022) healthcare provider competencies, including, for example, injunctions not to be discriminatory, to listen well, and to provide unbiased information.

Such research and associated recommendations are valuable and necessary, but they do not adequately address questions of the norms and power dynamics undermining person-centred care, including how these are integral to the systems themselves (Stojanovic et al., 2016). They fail to locate healthcare providers within the biomedicalised power relations underpinning users’ *and* providers’ experiences. The discourses of medicalised risk and biomedical expertise that we identified are not merely about ensuring contraceptive efficacy but also reflect entrenched paternalism (Stevens, 2015). Our findings here echo previous work from this study, which demonstrated how Aotearoa providers’ benevolent positioning and emphasis on preventing unintended pregnancy contribute to an enduring pattern of clinical paternalism, thereby normalising power imbalances in contraceptive care (Morison, 2022).

Alongside a range of systemic challenges (e.g. stock-outs), our participants’ narratives point to various disciplinary technologies – power relations inciting users and providers to particular discursive practices. These include the following practices in contraceptive healthcare encounters: (i) privileging healthcare providers’ biomedical knowledge; (ii) demeaning service users’ embodied knowledge; (iii) managing the risk of unintended pregnancies through inciting (‘selling,’ ‘promoting’) the use of contraception, in particular LARCs; and (iv) dividing practices that foreground particular people (low income, young people, people of colour) as particularly ‘vulnerable’ and in need of specific risk management interactions.

The discursive practices used to install these disciplinary technologies highlighted in our analysis include: (i) the confession (e.g. ‘tell me the truth’), (ii) responsibilisation (e.g. ‘you need to be able remember to take it [the pill] on time’), and (iii) surveillance (e.g. ‘We do look at your reliability in all the type of things’)—as echoed in a South African study (by the second author) on healthcare for pregnant teenagers (Macleod and Durrheim, 2002). These discursive practices are not just aspects of ‘provider bias’ or quality of care, but shape the conditions of possibility for provision and voluntariness that are understood and negotiated interpersonally and within particular systems of constraint (Nandagiri, 2021). Thus, while resource constraints could, in principle, be solved through careful financial planning and management, the discursive practices referred to above will persist if biomedicalised expertise and public health imperatives of managing unintended pregnancy risk continue to be prioritised.

Our analysis shows how the ideal of ‘responsible’ reproductive management – with its distinction between intended (‘planned’) and unintended (‘unplanned’) pregnancy – is fundamental to how biopower operates in contraceptive healthcare interactions. The discursive practices that we identified function to maintain an ideal of responsible reproductive management, ensuring the proper spacing, timing, and number of children. Those diverging from the ideal of responsible’ reproductive management can be positioned as irresponsible or at risk (or both), justifying intervention to discipline them as wayward reproductive subjects. In addition, biomedical experts’ power to intervene is extended by discourses of bio-medicalised risk and protectionism to include pre-emptive intervention: singling out ‘at-risk,’ ‘high risk,’ or ‘vulnerable’ women as needing more disciplinary and invasive technologies of reproductive governance (Clarke et al., 2003).

Our study has two associated implications. The first relates to the scope of public reproductive health research. Reproductive politics is a crucial yet often overlooked dimension of service provision, particularly in systems-focussed research. The broader field of scholarship on contraceptive care – and person-centred care more generally – also lacks engagement with the political dimensions of care. Our Foucauldian analysis of biopower demonstrates how expanding beyond a systems-focussed approach can yield deeper insights. As we showed, established and taken-for-granted concepts – such as ‘responsible reproductive management’ and even person-centred care – can inadvertently reinforce power imbalances. Recognising these dynamics allows for a more critical engagement with the structural forces shaping contraceptive care. The second implication concerns how contraceptive ‘success’ is defined. Currently, the prevention of unintended pregnancy is positioned as the ultimate goal – the Holy Grail – of contraceptive services. Yet, given that unintended pregnancies account for nearly 48% of pregnancies globally (Bearak et al., 2020), they are almost the norm rather than an aberration.

Both of these implications demand a critical interrogation of ingrained assumptions (at the policy and training levels) in contraceptive services. These include: (i) The assumption that the most clinically effective methods (e.g. LARC) are universally desirable; (ii) The view that unintended pregnancy or abortion represents a failure of care; (iii) Framing discontinuation or self-directed non-use of LARC (or any method) as a service failure; and (iv) The institutionalised belief that reproductive management – especially through high-efficacy contraception – is an unquestioned social and individual good, contributing to social development and empowerment for all. Crucially, these assumptions overlook the ‘dividing practices’ inherent in both subtle and overt coercive contraception strategies. These practices target certain populations as particularly ‘at risk,’ often along intersectional lines of race/ethnicity, class, ability, and socio-economic status. Such dynamics are deeply embedded in (neo)colonial power relations, shaping who is encouraged – or pressured – to use certain contraceptive methods (Nandagiri, 2021). Ultimately, to support *all* women’s bodily integrity, the goal of contraceptive services should not be simply increasing uptake of high-efficacy contraception. Instead, services must support contraceptive care that moves beyond both expert-led coercion *and* neoliberal responsibilising models of autonomy and prioritise bodily integrity, reproductive agency, and the right to make contraceptive choices free from coercion or systemic pressure.

## Notes

1. We use scare quotes here since not all who access contraceptive services are doing so to plan a family.

2. An intrauterine system (IUS) releases a progestogen hormone, while an intrauterine device (IUD) releases copper.

3. A category used in designate South Africans of mixed racial heritage including indigenous Khoisan and other African or Asian peoples, many of whom were enslaved at the Cape colony (See Alexander, 2022).

4. These participants requested a group interview as a cultural preference and to save time.
